# Efficacy of MP1032 in hospitalised patients with COVID-19— authors’ reply

**DOI:** 10.1016/j.lanepe.2024.100857

**Published:** 2024-02-02

**Authors:** Petra Sager, Astrid Kaiser, Sara Schumann, Beate Ludescher, Wolfgang Brysch

**Affiliations:** a MetrioPharm Deutschland GmbH, Am Borsigturm 100, 13507, Berlin, Germany; b MetrioPharm AG, Europaallee 41, Zurich, 8004, Switzerland

In their Correspondence letter, Liu et al.^1^ question whether the clinical trial's design, recently published in *The Lancet Regional Health—Europe* entitled Efficacy and safety of MP1032 plus standard-of-care compared to standard-of-care in hospitalised patients with COVID-19,^2^ was adequate in its population selection and timing of administration. In this study, the inclusion criteria allowed hospitalised patients with moderate or severe COVID-19, but not critically ill patients.

The first point raised by Liu et al. is that immune modulators, like JAK inhibitors and glucocorticoids, are primarily indicated in COVID-19 patients in severe or critical condition. While this is certainly true for these immune-suppressive drug classes, the case for MP1032 is different. As shown in Schumann et al.,^3^ the drug candidate MP1032 works as a scavenger for reactive oxygen species (ROS) acting directly and exclusively at the site of inflammation. Hence, in contrast to immune-suppressing biologics or glucocorticoids, MP1032 acts as immune-modulating drug that does not completely suppress cytokine induction, which is important during the initial innate anti-viral response. This unique pharmacodynamic profile therefore lends itself to the treatment of earlier stages of COVID-19. It was precisely this rationale that has guided the focus on moderate-to-severe, but non-critical patients as study population. In addition, it is crucial to halt the progression of the disease in its early (mild to moderate) stages and prevent the dreaded cytokine storm in order to reduce mortality and the burden on healthcare systems.

The second argument by Liu et al. points into the opposite direction, reasoning that a drug inhibiting the viral replication should be administered as early as possible after initial infection. Again, this is a valid point supporting our population selection in regard to moderate COVID-19 patients. As stated in the paper, the mean elapsed time since COVID-19 diagnosis was 3.2 days and 65.2% of patients were randomised within three days, which was relatively fast given the challenges of a clinical trial in a pandemic. Furthermore, so far unpublished preclinical experiments, which were also part of this Horizon Europe project, demonstrate that treatment with MP1032 led to a dose-dependent reduction of SARS-CoV-2 replication regardless of the time of addition, although the best anti-viral effect could be detected, when MP1032 was administered during and after an infection.

In conclusion, as a host-directed drug, MP1032 has promising anti-viral and immune–modulatory properties, positioning it as a viable candidate for the development of broad-spectrum anti-viral drugs in future early-intervention pandemic preparedness strategies.

## Contributors

AK, WB and PS decided to publish the response to the correspondence letter. AK, WB, BL, SS and PS provided input to the correspondence letter. WB, SS, BL drafted the letter and AK, PS critically revised the letter. All authors have read and agreed to the published version of the manuscript.

## Declaration of interests

Authors’ affiliations are as follows: PS, AK, SS, BL are employees of MetrioPharm Deutschland GmbH, a 100% affiliate of MetrioPharm AG. WB is employer, co-founder, and CSO of MetrioPharm AG and CMO of MetrioPharm GmbH.
